# Antimicrobial properties of *Limosilactobacillus reuteri* strains for control of *Escherichia coli* and *Salmonella* strains, diarrhoea cause in weaning pigs

**DOI:** 10.17221/112/2022-VETMED

**Published:** 2023-05-29

**Authors:** Yoonjeong Yoo, Jihwan Lee, Jinho Cho, Yohan Yoon

**Affiliations:** ^1^Department of Food and Nutrition, Sookmyung Women’s University, Seoul, Republic of Korea; ^2^Department of Poultry Science, University of Georgia, Athens, GA, USA; ^3^Department of Animal Sciences, Chungbuk National University, Cheongju, Republic of Korea; ^4^Risk Analysis Research Center, Sookmyung Women’s University, Seoul, Republic of Korea

**Keywords:** antibiotic resistance, feed additive, gut health, gut microbiota, probiotics

## Abstract

This study aimed to use lactic acid bacteria isolated from piglet faeces to develop probiotics, allowing for the effective control of *Escherichia coli* and *Salmonella*. Lactic acid bacteria were isolated from the faeces of suckling piglets and identified by 16S rRNA sequencing, then examined for haemolysis; gelatinase activity; and resistance to acid, bile, and pancreatin. The antimicrobial activity of selected lactic acid bacteria isolates was examined for 8 *E. coli* and 7 *Salmonella* strains. One-hundred and sixty-four lactic acid bacteria isolates were identified from 118 piglet faecal samples, and 13 lactic acid bacteria isolates were selected from analyses of haemolysis; gelatinase activity; and resistance to acid, bile, and pancreatin. Of the selected 13 lactic acid bacteria isolates, *Limosilactobacillus reuteri* PF20-3 and PF30-3 strains had the highest antibacterial activity against *E. coli* and *Salmonella*.

Excessive use of antibiotics in livestock farms to prevent disease and promote growth has resulted in problems such as residual antibiotics and antibiotic-resistant bacteria (Reardon 2015; Yang et al. 2022; Youn and Seo 2022). Consequently, restrictions and regulations on the use of antibiotics are being reinforced in several countries. According to statistics on domestic antibiotic sales in livestock, antibiotic usage in pigs was the highest among livestock animals in 2020 in Korea (507 000 kg) (MFDS 2022). Furthermore, more antibiotic-resistant bacteria were isolated from pig and poultry carcasses than from cattle during an investigation of antibiotic resistance involving examining livestock and carcasses in Korea (MFDS 2022). It is therefore a necessity to promote research on antibiotic alternatives, such as probiotics or antimicrobial additives (Andre et al. 2022).

Pigs have the second highest weaning-period mortality rate (Peltoniemi et al. 2021), considering the fact that piglets experience extreme stress when they are separated from the sows and have a diet change from breast milk to feed. This may lower their immunity and cause an imbalance in the intestinal microbiota, increasing the risk of piglet mortality as infected with pathogens, particularly in those caused by increased proportions of *Escherichia coli* and *Salmonella* and decreased proportions of *Bifidobacterium* and *Lactobacillus* in the gut (Arguello et al. 2019).

Instead of antibiotics, probiotics have become an effective way to treat diarrhoea in weaned piglets, and are thus defined as being “beneficial to the host animal by improving the balance of intestinal microbiota” (Fuller 1989). The preferred probiotic strains currently utilised for both humans and livestock are *Lactobacillus*, *Streptococcus* (*Enterococcus*), and *Bifidobacterium*. These strains are beneficial constituents of a healthy gut microbiota (Liao and Nyachoti 2017; Kwoji et al. 2021; Das et al. 2022).

Upon ingestion, beneficial bacteria dominate the intestine and either competitively hinder the colonisation of pathogenic bacteria or lower the intestinal pH to protect against pathogenic bacteria (Medellin-Pena et al. 2007). Various probiotics can be isolated from food and maternal milk, as beneficial bacteria such as *Lactobacillus* spp. and *Bifidobacterium* spp. are likely to be found in the intestines of suckling or weaning piglets, which could be transferred through milk from sows. Thus, the beneficial lactic acid bacteria can also be detected in the faeces of suckling or weaned piglets (Hou et al. 2015).

This study isolated lactic acid bacteria from suckling piglets and examined both their probiotic properties and antimicrobial activity against *E. coli* and *Salmonella,* which cause diarrhoea in weaned piglets.

## MATERIAL AND METHODS

### Isolation of lactic acid bacteria

Faecal samples (*n* = 118) were collected from suckling piglets from four pig farms in Republic of Korea. One gram of each sample was diluted with 9 ml of 0.1% buffered peptone water (Becton, Dickinson and Company, Franklin, NJ, USA) and streaked on de Man, Rogosa, and Sharpe (MRS) agar (Becton, Dickinson and Company, Franklin, NJ, USA). It was then incubated at 37 °C for 48 h in an atmosphere of 90% N_2_, 5% CO_2_, and 5% H_2_. Two or three different colonies were recovered and streaked on fresh MRS agar to obtain isolated single colonies. Each colony was subjected to 16S rRNA sequencing using the universal primers 27F (5'-AGAGTTTGATCMTGGCTCAG-3') and 1 492R (5'-GGTTACCTTGTTACGACTT-3'). Sequencing was performed by Bionics (Seoul, Republic of Korea). The 16S rRNA sequences of the strains were compared with those obtained from the National Center for Biotechnology Information.

### Confirmation of safety of piglet isolates

#### HAEMOLYSIS

The cultured lactic acid bacteria isolates were streaked on Columbia agar containing 5% sheep blood (bioMérieux, Marcy l’Etoile, France) and incubated at 37 °C for 24 h to assess their haemolytic characteristics, in accordance with the guidelines of the American Society for Microbiology (Buxton 2005). Strains that did not have distinct clear zones around the colonies were non-haemolytic strains, and strains with partially cleared and green-coloured zones around colonies were α-haemolytic. Lactic acid bacteria strains with clear transparent zones around the colonies were β-haemolytic (Hacioglu and Kunduhoglu 2021).

#### GELATINASE ACTIVITY

Isolated colonies on the MRS agar were inoculated into a nutrient gelatin medium (MBcell, Seoul, Republic of Korea). The medium was incubated at 37 °C for 4 days in an atmosphere of 90% N_2_, 5% CO_2_, and 5% H_2_. Gelatinase-producing *Staphylococcus aureus* ATCC25923 was the positive control.

### Resistance to acid, bile, and pancreatin

#### PREPARATION OF LACTIC ACID BACTERIA INOCULUM

Suspensions of each lactic acid bacteria isolate were prepared and stored at –80 °C. When required, 100 μl of each thawed suspension was inoculated into 10 ml of MRS broth and cultured at 37 °C for 24 h in an atmosphere of 90% N_2_, 5% CO_2_, and 5% H_2_. Each culture was then transferred to a 15-ml conical tube and centrifuged at 1 912 × *g* at 4 °C for 15 minutes.

The supernatant was discarded and the cell pellet was washed twice with phosphate-buffered saline (PBS; pH 7.4) comprising of 0.2 g KCl, 0.2 g KH_2_PO_4_, 8.0 g NaCl, and 1.5 g Na_2_HPO_4_·7H_2_O in 1 l of distilled water. The cell pellet was resuspended in PBS and diluted to 1 × 10^7^ cfu/ml.

#### ACID TOLERANCE

Each lactic acid bacteria inoculum (1 × 10^7^ cfu/ml) and MRS broth (pH 2.5) were mixed in a 1 : 1 ratio and incubated at 37 °C in a 90% N_2_, 5% CO_2_, and 5% H_2_ atmosphere. After 0 h and 3 h of incubation, 100 μl aliquots were serially diluted and plated on tryptic soy agar (TSA; Becton, Dickinson and Company, Franklin, NJ, USA). Viable cells were counted after 48 h of incubation at 37 °C.

#### BILE RESISTANCE

One-hundred microliters of each lactic acid bacteria inoculum (1 × 10^7^ cfu/ml) was inoculated into 10 ml MRS containing 0.3% porcine bile salt (Sigma-Aldrich, St. Louis, MO, USA) and incubated at 37 °C in a 90% N_2_, 5% CO_2_, and 5% H_2_ atmosphere. After 0 h and 24 h of incubation, 100 μl aliquots were serially diluted and plated on TSA. Viable cells were counted after 48 h of incubation at 37 °C.

#### PANCREATIN RESISTANCE

One-hundred microliters of each lactic acid bacteria inoculum (1 × 10^7^ cfu/ml) was inoculated into 10 ml PBS containing 0.1% pancreatin porcine pancreas (Sigma-Aldrich, St. Louis, MO, USA) and incubated at 37 °C in a 90% N_2_, 5% CO_2_, and 5% H_2_ atmosphere.

After incubation for 0 h and 4 h, 100 μl aliquot were serially diluted and plated on TSA. The bacterial cell counts were enumerated after 48 h of incubation at 37 °C.

### Analysis of antibacterial effect of isolates to *E. coli* and *Salmonella*

#### PREPARATION OF LACTIC ACID BACTERIA INOCULUM

Thirteen lactic acid bacteria strains were selected based on results from the haemolysis, gelatinase activity, acid, bile, and pancreatin resistance analyses. One-hundred microliters of each suspension was added to 10 ml MRS broth and incubated at 35 °C for 20 hours. The subcultures were centrifuged at 1 912 × *g* at 4 °C for 15 min and the cell pellets were washed twice with PBS. The cell pellets were resuspended in PBS, and the optical density at 600 nm (OD_600_) was adjusted to 1.0. Three microliters of each lactic acid bacteria inoculum was spot-inoculated on MRS agar and incubated at 35 °C for 24 hours.

#### PREPARATION OF *E. COLI* AND *SALMONELLA*

*E. coli* strains (KVCC-BA2000145, KVCC-BA-2000146, KVCC-BA2000147, KVCC-BA2000148, KVCC-BA2000149, KVCC-BA2000150, KVCC-BA2000151, KVCC-BA2000152), and *Salmonella* strains (KVCC-BA2000155, KVCC-BA2000156, KVCC-BA2000157, KVCC-BA2000158, KVCC-BA2000159, KVCC-BA2000160, and KVCC-BA-2000161) isolated from pigs were obtained from Korea Veterinary Culture Collection (KVCC). All strains were cultured in 10 ml of tryptic soy broth (TSB; Becton Dickinson and Company, Franklin, NJ, USA) at 37 °C for 24 hours. One hundred microliter aliquots of each culture were transferred to fresh 10 ml of TSB and incubated at 37 °C for 24 hours. The subcultures were centrifuged at 1 912 × *g* at 4 °C for 15 min and washed twice with 10 ml of PBS. The bacteria were diluted with PBS to obtain 1 × 10 cfu/ml.

#### AGAR DIFFUSION SPOT ASSAY

Aliquots of each *E. coli* and *Salmonella* strain (100 μl) were spot-inoculated on 10 ml Brain Heart Infusion agar (Becton, Dickinson and Company, Franklin, NJ, USA), and overlaid on the MRS agar. The plates were incubated at 37 °C for 24 h, and the diameter of the clear zone of growth inhibition that developed on each plate was measured.

### Statistical analysis

Each experiment was repeated three times for statistical analysis. Data were analysed with a general linear model of SAS^®^ University Edition (SAS Institute Inc., Cary, NC, USA). A significant difference in least-squares means between samples was determined using a pairwise *t*-test at α = 0.05.

## RESULTS AND DISCUSSION

### Probiotic strains isolated from piglet faeces

One-hundred and sixty-four lactic acid bacteria strains were isolated from 118 piglet faecal samples obtained from four farms. Of the isolates, the majority were *Limosilactobacillus reuteri* (*n* = 76), followed by *Limosilactobacillus mucosae* (*n* = 20), *Streptococcus pasteurianus* (*n* = 14), *Ligilactobacillus salivarius* (*n* = 9), and *Enterococcus faecalis* (*n* = 3). In many countries, there are standard guidelines for the addition of lactic acid bacteria to food and feed. Thus, of the 164 lactic acid bacteria isolates, 87 isolates were chosen based on the Korean feed code (Ministry of Agriculture, Food and Rural Affairs 2022) for further analysis. Lactose is abundant in the mature milk of sows consumed by lactating piglets, and stimulates the proliferation of lactic acid bacteria, such as *Limosilactobacillus* (Zhao et al. 2021). *L. reuteri* and *L. salivarius* in particular are beneficial bacteria that are abundant in piglet intestines (Martin et al. 2009).

### Haemolysis and gelatinase activity of isolates

The absence of haemolytic activity is considered a prerequisite for the selection of probiotic strains as it ensures that opportunistic virulence will not appear among strains (FAO/WHO 2002). Of the 87 lactic acid bacteria isolates, 53, 10, and 24 isolates were judged to be α-haemolytic, β-hemolytic, and non-hemolytic, respectively. Fifty-two isolates of *L. reuteri* showed α-haemolysis, and 24 γ-haemolysis. Eight isolates of *L. salivarius* were β-haemolytic and one was α-haemolytic. Two isolates of *E. faecium* were β-haemolytic. Thus, only 24 γ-haemolytic isolates were analysed for gelatinase activity. Gelatinase is a proteolytic enzyme found in the connective tissues, and can act on gelatine, collagen and haemoglobin. It is also regarded as a pathogenic factor in probiotics as it degrades membrane components (Gupta and Sharma 2017; Oruc et al. 2021). In this test, the medium inoculated with *S. aureus* ATCC25923 was the positive control, and the uninoculated medium was the negative control. Of the 24 isolates, only *L. reuteri* PF58-2 was gelatinase-positive, and the remaining 23 gelatinase-negative and non-haemolytic strains may be considered safe.

### Acid, bile, and pancreatin resistance of isolates

According to Yasmin et al. (2020), lactic acid bacteria should be able to survive in the digestive system to reach the small intestine, where they colonise and provide the host with health benefits.

Acid resistance was examined for the 23 strains that were non-haemolytic and lacked gelatinase activity. After 3 h of incubation at pH 2.5, the viability of the *Lacticaseibacillus rhamnosus* (LGG) positive control was 90.9%. Except for *L. reuteri* PF51-1, the other 22 strains displayed survival rates of 43.3% to 119.3%, and showed no significant difference in acid resistance compared to the positive control (Table 1).

**Table 1:** Acid, bile, and pancreatin resistance of lactic acid bacteria

Strain	Acid resistance (%)	Bile resistance (%)	Pancreatin resistance (%)
LGG	90.9 *±* 40.7^abc^	53.5 *±* 59.1^ef^	62.8 *±* 28.1^c^
PF18-3	81.7 *±* 62.6^abc^	9.6 *±* 14.4^ef^	182.3 *±* 127.3^a^
PF19-1	67.4 *±* 30.4^abc^	18.7 *±* 26.7^ef^	120.4 *±* 119.5^abc^
PF20-3	86.2 *±* 49.4^abc^	33.6 *±* 45.3^ef^	110.3 *±* 54.5^abc^
PF21-2	72.1 *±* 42.5^abc^	31.4 *±* 27.8^ef^	134.5 *±* 89.3^abc^
PF22-2	84.1 *±* 38.7^abc^	108.8 *±* 95.0^cdef^	62.4 *±* 42.0^c^
PF25-1	78.6 *±* 49.5^abc^	6.6 *±* 11.1^ef^	128.1 *±* 68.3^abc^
PF27-1	72.8 *±* 29.4^abc^	0.0 *±* 0.0^f^	151.6 *±* 73.8^ab^
PF29-1	72.7 *±* 57.3^abc^	5.0 *±* 7.5^ef^	118.9 *±* 79.1^abc^
PF30-3	64.3 *±* 35.2^abc^	148.0 *±* 156.4^cde^	128.1 *±* 102.2^abc^
PF31-2	119.3 *±* 98.6^a^	21.9 *±* 41.0^ef^	60.4 *±* 17.4^c^
PF34-2	55.8 *±* 35.7^bcd^	217.0 *±* 262.9^bcd^	90.9 *±* 52.0^bc^
PF40-1	70.1 *±* 47.8^abc^	19.9 *±* 18.0^ef^	93.1 *±* 54.9^bc^
PF41-1	90.2 *±* 85.3^abc^	308.0 *±* 179.7^bcd^	122.0 *±* 61.8^abc^
PF43-1	101.9 *±* 63.8^abc^	328.0 *±* 203.5^a^	83.3 *±* 79.2^bc^
PF44-2	67.8 *±* 33.6^abc^	9.9 *±* 6.9^ab^	169.8 *±* 116.6^a^
PF45-1	84.0 *±* 58.2^abc^	210.7 *±* 198.6^ef^	92.1 *±* 50.9^bc^
PF46-1	47.9 *±* 33.8^cd^	402.3 *±* 81.9^a^	146.4 *±* 86.5^abc^
PF47-2	85.3 *±* 51.3^abc^	337.7 *±* 172.4^ab^	124.2 *±* 97.9^abc^
PF49-2	77.8 *±* 48.0^abc^	63.7 *±* 82.7^ef^	76.1 *±* 31.2^bc^
PF51-1	0.0 *±* 0.0^d^	0.1 *±* 0.1^f^	92.8 *±* 61.7^bc^
PF52-1	43.3 *±* 33.5^cd^	237.8 *±* 231.8^abc^	81.5 *±* 61.3^bc^
PF56-2	112.2 *±* 54.3^ab^	85.1 *±* 89.3^def^	78.6 *±* 40.4^bc^
PF94-2	87.6 *±* 43.3^abc^	130.6 *±* 219.6^cdef^	60.9 *±* 27.7^c^

^a–f^Different letters indicate a significant difference within columns (*P *< 0.05)

For the bile acid resistance, the survival rates of all 23 isolates were 0.02% to 402.3%. The isolates showed either excellent or no significant difference compared to LGG (Table 1). To confirm resistance to the digestive enzymes, the isolates were cultured in a medium containing porcine pancreatin. After 4 h of incubation, the viability of the positive control was 62.8%. All 23 lactic acid bacteria isolates displayed viability rates ranging from 60.4% to 182.3%. These survival rates were either superior to or not significantly different from LGG in digestive enzyme resistance (Table 1).

### Growth inhibition of *E. coli* and *Salmonella* by lactic acid bacteria isolates

**Figure 1:**
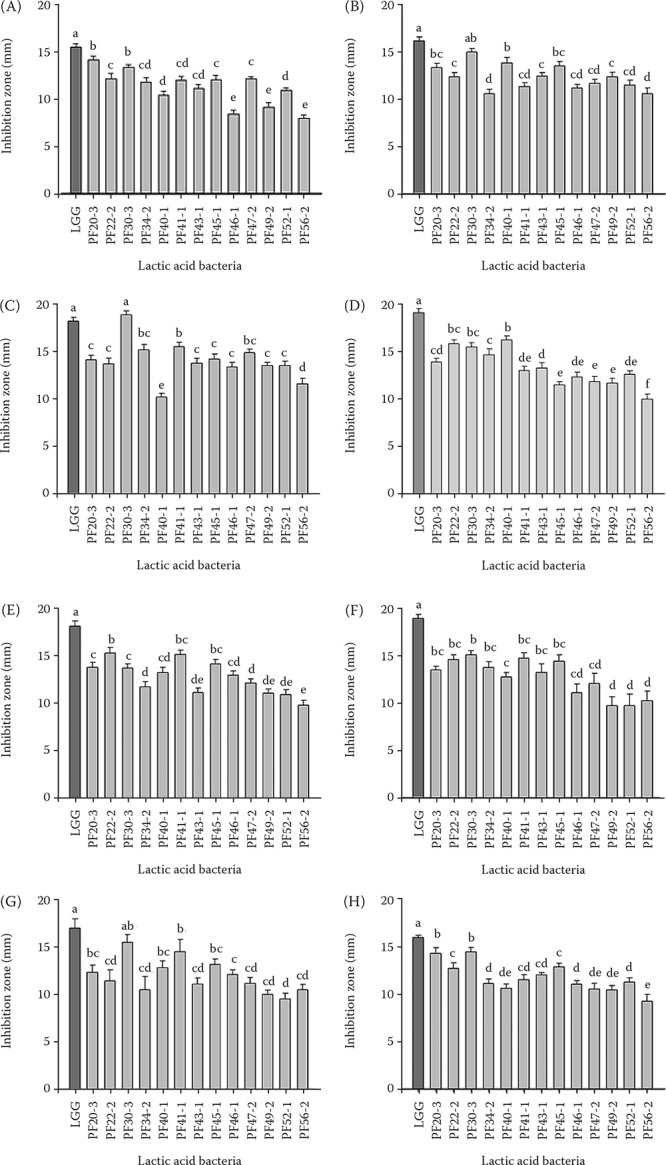
Antimicrobial activity of lactic acid bacteria isolates to *Escherichia coli* strains (A) KVCC-BA2000145, (B) KVCC-BA2000146, (C) KVCC-BA2000147, (D) KVCC-BA2000148, (E) KVCC-BA2000149, (F) KVCC-BA2000150, (G) KVCC-BA2000151, (H) KVCC-BA2000152 ^a–f^Different letters indicate a significant difference (*P *< 0.05)

**Figure 2:**
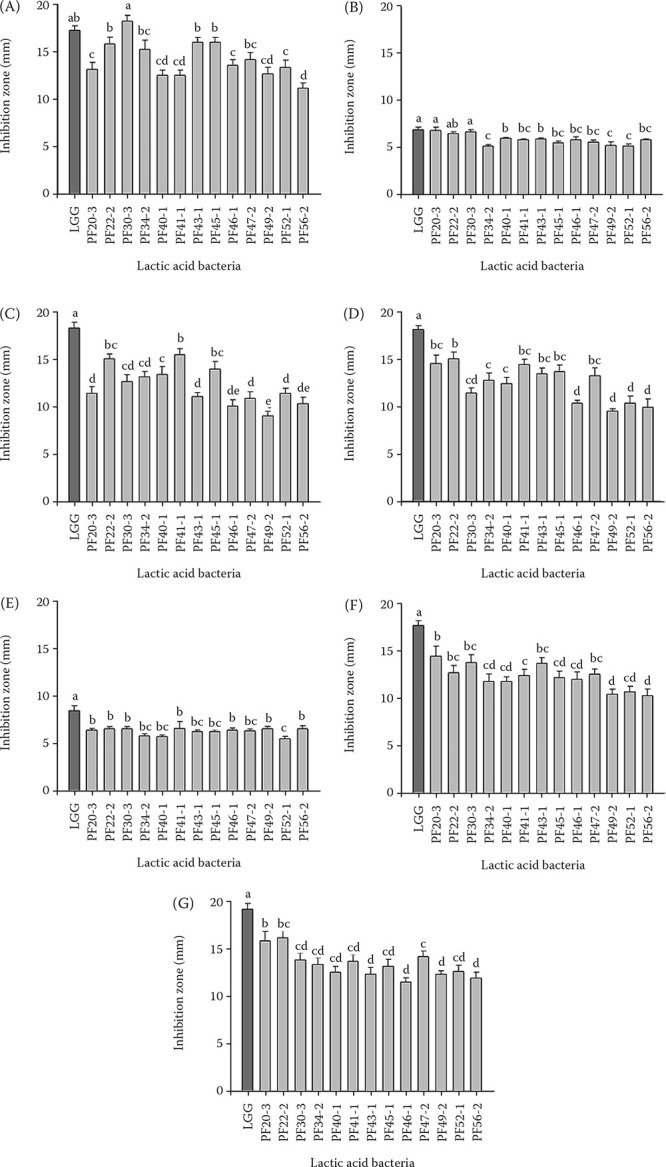
Antimicrobial activity of lactic acid bacteria isolates to *Salmonella* (A) KVCC-BA2000155, (B) KVCC-BA2000156, (C) KVCC-BA2000157, (D) KVCC-BA2000158, (E) KVCC-BA2000159, (F) KVCC-BA2000160, and (G) KVCC-BA2000161 ^a–e^Different letters indicate a significant difference (*P *< 0.05)

*L. reuteri* strains PF20-3 and PF30-3 displayed the highest antimicrobial activity against *E. coli* and *Salmonella* strains (Figures 1 and 2).

PF30-3 had a large inhibitory effect on 11 of the 15 strains of diarrhoeal pathogenic bacteria, and PF20-3 had an outstanding inhibitory effect on 10 of 15 strains. Controlling *E. coli* and *Salmonella* is as important as the management of diarrhoea in weaned pigs, and it can result in death, increasing economic losses (Barba-Vidal et al. 2018; Burdick Sanchez et al. 2019). *Lactobacillus*, a type of lactic acid bacteria used in probiotics, has the ability to destroy pathogenic bacteria by producing organic acid such as lactic acid, and bacteriocin (Bilkova et al. 2011). Lactic acid is transferred to the cytoplasm of bacteria and lowers the intracellular pH, as well as reacting with the cell membrane and causing protein denaturation (Huang et al. 1986; Zare Mirzaei et al. 2018). Bacteriocin first increases the permeability of the cytoplasmic membrane, dissipates the proton motive force, degrades vital macromolecules such as DNA and RNA, and causes cell lysis (Christensen and Hutkins 1992; Montville and Bruno 1994). In these modes, *L. reuteri* strains PF20-3 and PF30-3 may show an antimicrobial effect against *E. coli* and *Salmonella.*

In conclusion, it could be stated that our collective findings indicate the potential of the isolated *L. reuteri* strains PF20-3 and PF30-3 as probiotic strains that can inhibit *E. coli* and *Salmonella*, which can cause diarrhoea in weaning piglets. Thus, these strains need to be further examined in an animal model.
